# Brain size and brain/intracranial volume ratio in major mental illness

**DOI:** 10.1186/1471-244X-10-79

**Published:** 2010-10-11

**Authors:** Martin Reite, Erik Reite, Dan Collins, Peter Teale, Donald C Rojas, Elliot Sandberg

**Affiliations:** 1 Department of Psychiatry, University of Colorado Denver, Aurora CO, USA; 2 Eglin AFB Hospital, Ft Walton Beach, FL, USA; 3 Radiology Department, Denver VAMC, Denver, CO, USA

## Abstract

**Background:**

This paper summarizes the findings of a long term study addressing the question of how several brain volume measure are related to three major mental illnesses in a Colorado subject group. It reports results obtained from a large N, collected and analyzed by the same laboratory over a multiyear period, with visually guided MRI segmentation being the primary initial analytic tool.

**Methods:**

Intracerebral volume (ICV), total brain volume (TBV), ventricular volume (VV), ventricular/brain ratio (VBR), and TBV/ICV ratios were calculated from a total of 224 subject MRIs collected over a period of 13 years. Subject groups included controls (C, N = 89), and patients with schizophrenia (SZ, N = 58), bipolar disorder (BD, N = 51), and schizoaffective disorder (SAD, N = 26).

**Results:**

ICV, TBV, and VV measures compared favorably with values obtained by other research groups, but in this study did not differ significantly between groups. TBV/ICV ratios were significantly decreased, and VBR increased, in the SZ and BD groups compared to the C group. The SAD group did not differ from C on any measure.

**Conclusions:**

In this study TBV/ICV and VBR ratios separated SZ and BD patients from controls. Of interest however, SAD patients did not differ from controls on these measures. The findings suggest that the gross measure of TBV may not reliably differ in the major mental illnesses to a degree useful in diagnosis, likely due to the intrinsic variability of the measures in question; the differences in VBR appear more robust across studies. Differences in some of these findings compared to earlier reports from several laboratories finding significant differences between groups in VV and TBV may relate to phenomenological drift, differences in analytic techniques, and possibly the "file drawer problem".

## Background

This paper addresses differences in several measures of brain and ventricle volume and brain/intracranial volume ratio in three major Axis I mental disorders including schizophrenia (SZ), schizoaffective disorder (SAD), and bipolar disorder (BD), based upon MRIs of the brain obtained from 224 subjects over a period of 13 years in the same laboratory. Originally obtained for the purpose of providing brain structural data for neuroanatomical source location of MEG determined functional sources, this MRI data base is now being examined from a strictly anatomical volumetric viewpoint, to compare data from this subject population to similar reports in the published literature to date.

Brain size and the ratio of brain size to total intracranial volume has been a topic of interest since the advent of the capacity to image the brain. The earliest imaging strategy, pneumoencephalography, was introduced by Walter Dandy, chief resident for William Halstead at Johns Hopkins, in 1919, replacing cerebral spinal fluid (CSF) with air, which made it possible to study the contours and major morphological changes in the brain directly [1]. Abnormalities in the earliest studies in patients with dementia and the organic psychoses, led Moore et al suggested in 1935 that if similar changes could be demonstrated in patients with the so-called "functional psychoses" it would imply disturbances in brain function also underlying these disorders [2]. These authors reported PEG result in 71 patients with schizophrenia and 46 patients with manic depressive psychosis. Evidence of cortical atrophy was found "in the majority of patients with schizophrenia" (p57), but in the cases of manic depressive psychosis these investigators stated "The encephalograms in this group showed no consistent picture that would characterize manic-depressive psychosis" (p61). Haug in 1962 [3] reviewed PEG studies of schizophrenia to date, and added 101 new cases of schizophrenia, of which 73 had a diagnosis of definite or probable dementia as well, finding evidence of abnormal PEGs in 58%, usually ventricular dilatation or increased subarachnoid space suggestive of cortical atrophy. In general, the early PEG studies were complicated by relative lack of diagnostic clarity, absence of controls, and the fact that patient populations were most often chronically hospitalized and frequently demented individuals with many co morbidities, as well as poor resolution and difficulty quantifying the imaging data.

The development of computerized axial tomography greatly enhanced the capacity to visualize the outlines of the brain and ventricular system and identify significant structural abnormalities, although volumetric calculations were compromised by issues of slice thickness, and difficulties estimating the volume of radiolucent CSF (e.g. in the sulci). A review of 50 CT studies in schizophrenia reported inconsistency (and diminution) of findings over time, and the interesting observation that studies in larger numbers of subjects appeared to less often find significant differences compared to studies with fewer subjects [4].

The subsequent development of magnetic resonance imaging (MRI), in association with the dramatic increase in computational capabilities including computerized image analysis, led to an explosion of neuroanatomical studies of brain structure in mental illness. As of the date of this writing, a Medline search combining CT, brain and schizophrenia retrieve 443 publications, and brain, schizophrenia, and MRI return 1152 publications. In the case of bipolar disorder, searches of bipolar disorder and manic depressive disorder, CT, and brain return 70 publications, and with MRI instead of CT, 228. Salient is the development of major data bases such as the 'Internet Brain Volume Database'[5] funded by 'The Human Brain Project' which attempts to archive this extensive volumetric data.

SZ, now generally considered to represent a neurodevelopmental disorder, has been studied most intensively in terms of brain volume changes. Findings were often not consistent however. Earlier studies frequently suggested fairly significant volumetric differences in patients compared to controls; later studies usually with larger Ns have often been more equivocal. In a 1999 review of 8 longitudinal MRI studies of brain structural changes in SZ (which included a number of structures as well as ventricle size), DeLisi [6] was only able to conclude that changes in such variables appear greater across the life span in subjects with SZ compared to controls, but the specifics are highly variable.

The brain volume of patients with BD has been less intensively studied.

A meta-analysis published by McDonald et al in 2004 systematically analyzed twenty six studies which investigated volumetric measurement on up to 404 BD patients [7]. Their conclusions established that the volumes of most brain structures are preserved in BD other than a noted association with right-sided ventricular enlargement.

No studies yet independently report brain volume or brain/ICV ratio in SAD, which seems unusual for a disorder which, at least in the Denver public mental health system, outnumbers SZ in frequency. There is no independent MESH code for SAD, and when used as a keyword, it is rather included under the terms schizophrenia and disorders with psychotic features, perhaps related to sparsity of published biomarkers specific to SAD.

This manuscript reports the findings from this group of subjects addressing several areas, including 1) how replicable is the evidence supporting altered brain volume (BV) in these major mental disorders, 2) is there evidence supporting altered intracranial volume (ICV, the space available for the brain to fill) in these disorders, 3) what is the evidence for altered ratios of BV to ICV, suggesting BV may have changed after ICV developed, and 4) what is the evidence for altered VV and VBR in these disorders.

The manuscript is based upon data collected with the support of several NIH grants over approximately the past 13 years, which offers advantages (relatively large number of subjects, methodological consistency within the same laboratory), and of course some possible problems (imaging equipment changes with time).

## Methods

### Subjects

We obtained MRI scans from a total of 224 subjects over a time period of thirteen years, beginning in 1992. Subjects were participants in one or more of two NIMH funded R01 grants studying MEG based biological variables in mental disorders, and included individuals with SZ (N = 58, 40 males), SAD (N = 26, 18 males), BD (N = 51, 24 males), as well as normal controls (C, N = 89, 42 males).

Patient subjects of any race between the age of 18 and 58 that met the DSM-IV criteria for BD, SAD or SZ that were without the presence of a current or recent (past 3 mo) diagnosis of alcohol or substance abuse/dependence, had no history of a neurological disorder (epilepsy, stroke, traumatic brain injury, significant environmental/toxic injury, other neurodevelopmental or neurodegenerative disorders, past meningitis/encephalitis, autism, pervasive developmental disorder, or mental retardation), or current major medical illness were eligible for the study. All patient subjects were recruited from the Denver metropolitan area and were in outpatient treatment. Psychiatric diagnoses were based upon a formal structured diagnostic interview (SCID-P) performed by MR or a research assistant that had been trained to criteria on SCID interview procedures with review of SCID findings with MR. Comparison control subjects were community volunteers with no history of mental illness or neurological disease. Control subjects met criteria for never mentally ill on the SCID-NP. All participants completed the Annett Handedness Scale[8].

The majority of patient subjects were medicated. Most SZ subjects were taking typical or atypical antipsychotics, most BD patients taking mood stabilizers as well as possibly antipsychotics, and SAD patients taking various combinations of mood stabilizers and antipsychotics. Demographic and medication data for the all subjects are summarized in Table 1.

**Table 1:** Group demographics.

Characteristic	Bipolar group	Schizoaffective group	Schizophrenic group	Controls
Number of subjects	51 (24 males)	26 (18) males	58 (40 males)	89 (42 males)
Age (std dev)	40.65 (10.85)	36.37 (11.78)	39.22 (7.95)	34.34 (8.79)
Education years	14.45 (2.03)	13.30 (2.42)	12.94 (2.55)	15.26 (1.91)
Handedness (Annette score)	0.85 (0.14)	0.85 (0.27)	0.71 (0.49)	0.79 (0.36)
Number medicated	45	24	54	0

All experimental protocols were approved by the Colorado Multiple Institutional Review Board, and after the studies had been fully explained to them, all subjects were required to sign an informed consent. BD subjects were studied in a euthymic state, as defined by a Hamilton Depression Rating Scale score < 7, and Young Mania Rating Scale score < 6.

### MRI Data Acquisition

MRIs were obtained at one of three sites: including a GE Signa 1.5 T (153 scans) scanner at the University of Colorado Hospital, a 1.5 T Philips NT (48 scans) scanner at the Denver VAMC, and a GE 3.0 T (23 scans) MRI scanner located within the Department of Psychiatry, UCDenver. Standardized T1 weighted image protocols (TR = 40 ms, TE = 5 ms) were used on all instruments, imaging the head with 124 1.7 mm thick, contiguous coronal images, voxel dimensions 0.94 × 0.94 mm × 1.7 mm. The proportion of scans across the 3 scanners among the 4 groups was not significantly different, χ^2 ^(6) = 11.12, p > .05.

A single investigator (ER) determined all intracranial and brain volumes over the total course of the study. Formal training in brain volume identification including accurate delineation of the skull-CSF boundary was provided by a board certified neuroradiologist (ES). A combination of manual and automated brain extraction techniques based upon IDL software[9] was used to identify and extract the intracranial volume and brain volume contained within. Briefly, each slice in the coronal series was displayed on the computer screen, and an initial computer estimate of inner skull boundary, CSF, and brain tissue in that slice based upon pixel intensity values was performed automatically using the contour-based thresholding function of IDL. Each resulting slice with automated estimates was then visually examined sequentially, slice by slice, in detail. The accuracy of the inner skull border was determined visually, necessary corrections were made using hand tracing, and the resulting bone and tissue external to this boundary was stripped leaving ICV containing brain and CSF for that slice. Next the estimate of CSF - brain boundary was examined and corrected visually by hand as necessary, and CSF in that slice was removed, leaving brain tissue for that slice. These functions were performed sequentially for each brain MRI slice from front to back. The entire procedure required approximately 3-4 hours for each brain. A more detailed comment on methods for identifying ICV boundaries can be found in appended Additional file 1.

Additionally, subsequent processing was used to independently separate ventricular from non-ventricular CSF based upon several automated methods. Using FSL "Fast" segmentation software [10], the brains (which had already had all tissue external to the CSF-inner table boundary removed) were segmented and the three tissue types, grey, white and csf were classified by pixel value. Using high-dimensional warping software "Hammer" [11] the images were warped to a ventricle labeled brain template. Individual subjects image volumes were then multiplied by the inverse of the deformation field retained from the warp into template space, resulting in ventricle volumes for each subject in their original space. A ratio of brain volume (with ventricular volume removed) to intracranial volume (TBV/ICV), and ventricle/brain ratio (VBR) was then computed for each subject.

Statistica 6.1 (Statsoft, Tulsa, OK) software was used for data analysis. Null-hypothesis significance testing was conducted at .05 alpha (two-tailed), using Type III sums of squares. Differences in demographic variables between groups were evaluated using separate one-way, between groups ANOVA. The effect of scanner on MRI measures was assessed using one-way ANOVAs. To examine the impact of gender on the MRI variables, Independent Student's t-tests were computed separately for the dependent measures. To evaluate group effects for the MRI variables, a one-way ANCOVA was conducted separately for total brain volume (TBV), ventricular volume (VV), intracranial volume (ICV) and the ratio of brain volume to intracranial volume, using gender and age as covariates for the analyses. Pearson Product Moment Correlation Coefficients were used to compute correlations between demographic variables and MRI variables. Post-hoc analyses of group main effects were conducted using Fisher's Least Significant Difference (LSD) tests. A one-way ANOVA as used to examine VBR and diagnosis as the between subjects factor.

## Results

A summary of mean vales and standard deviations for ICV, TBV, VV, VBR and TBV/ICV ratio are tabulated in Table 2.

**Table 2:** Means and standard deviations (SD) for intracranial volume (ICV), total brain volume (TBV), ventricular volume (VV), ventricle/brain ratio (VBR), and brain volume/intracranial volume ratio (TBV/ICV).

Bipolar Subjects		ICV	TBV	VV	VBR	TBV/ICV
**Male (n = 24)**	Mean ± SD	1482.563 ± 138.828	1329.843 ± 129.378	31.51 ± 13.9	0.243 ± 0.0102	0.897 ± 0.030
**Female (n = 27)**	Mean ± SD	1302.536 ± 112.330	1166.931 ± 110.786	21.77 ± 6.08	0.0192 ± 0.0056	0.895 ± 0.019
**Total (n = 51)**	Mean ± SD	1387.255 ± 153.827	1243.596 ± 144.313	26.64 ± 10.05	0.0217 ± 0.0079	0.896 ± 0.025
**Control Subjects**		**ICV**	**TBV**	**VV**	**VBR**	**TBV/ICV**
**Male (n = 42)**	Mean ± SD	1489.755 ± 114.978	1354.338 ± 111.556	25.18 ± 9.90	0.0190 ± 0.0075	0.908 ± 0.018
**Female (n = 47)**	Mean ± SD	1345.118 ± 116.599	1215.653 ± 105.602	20.99 ± 6.25	0.0176 ± 0.0048	0.903 ± 0.021
**Total (n = 89)**	Mean ± SD	1413.374 ± 136.157	1281.100 ± 128.356	23.08 ± 8.07	0.0182 ± 0.0061	0.906 ± 0.020
**Schizoaffective Subjects**		**ICV**	**TBV**	**VV**	**VBR**	**TBV/ICV**
**Male (n = 18)**	Mean ± SD	1435.895 ± 118.635	1298.308 ± 101.764	26.33 ± 10.96	0.0208 ± 0.0084	0.904 ± 0.016
**Female (n = 8)**	Mean ± SD	1328.658 ± 122.739	1196.083 ± 107.790	24.71 ± 4.40	0.0213 ± 0.0046	0.900 ± 0.015
**Total (n = 26)**	Mean ± SD	1402.899 ± 127.814	1266.854 ± 112.296	25.52 ± 7.68	0.0210 ± 0.0065	0.903 ± 0.015
**Schizophrenic Subjects**		**ICV**	**TBV**	**VV**	**VBR**	**TBV/ICV**
**Male (n = 40)**	Mean ± SD	1464.978 ± 126.294	1315.718 ± 118.423	29.15 ± 0.36	0.0227 ±0.073	0.898 ± 0.018
**Female (n = 18)**	Mean ± SD	1263.270 ± 123.169	1136.627 ± 133.719	24.24 ± 8.66	0.0217 ± 0.0068	0.898 ± 0.027
**Total (n = 58)**	Mean ± SD	1402.379 ± 158.788	1260.138 ± 148.032	26.69 ± 9.01	0.0222 ± 0.0071	0.898 ± 0.021

Volumes in ml.

TBV, ICV and VBR did not significantly differ between scanners. Given that and the lack of significantly different proportions of patient groups between the scanners, the scanner variable was not considered further in subsequent analyses.

There were significant gender differences in all of the volume measurements, but not for the TBV/ICV ratio measure. For VV (not illustrated), TBV and ICV, men had significantly larger volumes than women, t(222) = 4.63, p < .001, t(222) = 8.98, p < .001 and t(222) = 9.38, p < .001, respectively. There was a significant difference in age between groups, F(3, 220) = 6.02, p < .001. Post-hoc analyses revealed that the C group (mean age 34.34 years) was significantly younger than the BD (40.65 years) and SZ (39.22 years) groups, p < .001 and p = .002 respectively. Age was significantly correlated with VV (r = .25, p < .001), TBV (r = -.15, p < .05) and TBV/ICV ratio (r = -.19, p = .005), but not with ICV (p = -.12, p = .08). We therefore employed both age and gender as covariates in subsequent analyses.

For TBV, the group main effect, although trending, was formally statistically non-significant, F(3, 218) = 2.42, p = .07. Likewise, for ICV the group main effect was non-significant, F(3, 218) = 1.62, p = .19. No group differences in VV were observed, F(2, 218) = .81, p = .49. For the TBV/ICV measure, the group main effect was however significant, F(3,227) = 2.58, p = .05. Post hoc analysis revealed that the TBV/ICV ratio in both BD and SZ subjects were smaller than controls, p = .007 and p = .005 respectively.

The ANOVA for VBR found that the diagnosis main effect was significant, F(3,220) = 4.74, p = .003. Posthoc LSD testing revealed that the BD and SZ groups had significantly higher ratios than controls (p = .009 and p = .001), but theSAD group was not significantly different than C (p >.05). No other effects were significant.

Examination of the raw mean values for several of the variables might suggest concordance with recently published data for SZ. The SZ patients indeed demonstrated smaller brains. The male SZ subjects had TBV 38 cc (about 3%) smaller than male controls; females with SZ had TBV 79 cc (about 6%) smaller than controls. ICV values were also slightly smaller in the SZ groups however. None of these differences reached formal statistical significance however reflecting intrinsic variability in the measures. In the bipolar group, BP males had BV 8 cc larger than controls; BP females had brains 43 cc smaller than controls, and ventricular volumes were not different. Both male and female schizoaffective subjects had smaller raw mean BV than controls, but again the differences were not significant statistically, and their slightly smaller ICVs led to their BV/ICV ratios being essentially identical to controls. Their VV did not differ from controls. It is clear that rigorous statistical control exerts significant influence on the interpretation of means such as these in such subject cohorts.

We have included additional figures (Additional files 2, 3, 4 &5) containing raw data sets which illustrate the relationship of values for 1) brain volume, 2) ventricle/brain ratio, 3) ventricular CSF volume, and 4) brain/ICV ratio to age.

## Discussion

Several issues must be considered as we discuss these findings. First might be how do our absolute values compare with previous published findings in the medical literature for these subject groups. For comparison, we chose recent publications utilizing thin contiguous MRI slices.

### Comparisons of control volumes

With respect to control subjects, we examined how our values for TBV and ICV compare to TBV and ICV values extracted from 5 other published studies involving 243 normal subjects (comparison studies include those of Tanskannen [12], Narr [13], Arango [14], Matsumae[15], and Blatter [16] These comparisons are illustrated in Table 3.

**Table 3:** Comparison charts for ICV and TBV - all in ml.

Author	Age range	Control male ICV	Control female ICV	Control male TBV	Control female TBV
Reite et. al. this ms.	18-55	(N = 42) 1490 ± 115	(N = 47) 1345 ± 117	1354 ± 111	1216 ± 106
Tanskannen et. al.2009	33-35	(N = 60) 1150 ± 114	(N = 40) 1378 ± 91	1351 ± 101	1215 ± 88
Narr et.al. 2003	33-35	(N = 15) 1363 ± 135	(N = 13) 1244 ± 89	1273 ± 129	1168 ± 81
Arango et.al. 2008	33-35	(N = 34) 1545 ± 133	(N = 32) 1333 ± 95	1424 ± 137	1220 ± 91
Matsumae et.al. 1996	24-80	(N = 26) 1469 ± 102	(N = 23) 1289 ± 111	1302 ± 112	1143 ± 105
Blatter et.al. 1996	36-45	(N = 17) 1546 ± 104	(N = 23) 1358 ± 113	1407 ± 99	1246 ± 105
Mean of means		1494	1324	1352	1201

Our ICV and TBV means for both males and females were contained within the range of the means of these studies. Our ICV values differed by 0.3% in males, and 1.6%in females; for TBV our results differed by 0.2% in males, and 1.2% in females. All in all therefore, we believe the ICV and TBV in the control subjects in our study compare favorably with those reported by other investigators.

### Comparisons of schizophrenia volumes

We compared our findings in patients with schizophrenia to published values in 3 other recent studies reporting both BV and ICV in schizophrenia, those of Narr, [13], Arrango, [14], and Tanskanen [12]. These comparisons are illustrated in Table 4.

**Table 4:** Comparison charts for schizophrenic (Sz) volumes - all in ml.

Author	Age range	Sz maleICV	Sz female ICV	Sz male TBV	Sz female TBV
Reite et al, this ms.	18-55	(N = 40) 1465 ± 126	(N = 18) 1263 ± 123	1316 ± 118	1137 ± 134
Tanskannen et.al. 2009	33-35	(N = 31) 1354 ± 125	(N = 23) 1365 ± 79	1328 ± 110	1182 ± 73
Narr et.al. 2003	33-35	(N = 15) 1380 ± 118	(N = 10) 1237 ± 114	1268 ± 110	1152 ± 97
Arango et.al. 2008	33-35	(N = 64) 1507 ± 154	(N = 21) 1332 ± 119	1365 ± 142	1209 ± 115
Mean of means		1426	1299	1319	1170

Our values for both ICV and TBV are quite comparable with these other published values. The standard deviation in the several studies are all quite similar, and generally large - in the vicinity of 100-150 cc or about 8-12% of total brain volumes. For illustrative purposes, the mean of the means are also tabulated for comparison with individual study values.

Harrison in a 1999 review of the neuropathology of schizophrenia comments that despite over a hundred years of research on the topic, specifics remain obscure, with studies using meta-analyses most often supporting evidence of increased ventricular volume and selected decreases (cortex and hippocampus) in brain volume [17]. Interestingly however, in the Harrison meta-analysis this difference did not emerge until the 50-60yo age group of men, and was equivocal in women before the age of 70, and our subject population was younger.

A meta-analysis by Woods and colleagues utilized data from 20 publications addressing ICV and TBV in SZ, involving a total of 1049 controls and 982 patients with TBV data, and 942 controls and 889 patients with extra cerebral volume (ECV). SZ patients demonstrated a TBV reduction of 34cc, and ECV increase of 14.1cc [18]. These differences, while statistically significant, were small, pointing out that a very large N is necessary to establish such small differences as being significant. With brain volumes generally in the 1200-1400cc range, and standard deviations in the range of 100cc, a difference of 34cc represents about 3% of total TBV, or about one third of one typical standard deviation.

In light of two large meta-analyses reporting similar but quite small differences in TBV between NC and SZ patients, the question arises of why the large majority of early published studies utilizing relatively small Ns quite frequently reported statistically significant differences in relatively small subject groups. One issue is possible phenotypic drift, wherein the type of patient included in a given diagnostic cohort changes over time. Certainly the chronically hospitalized and non-medicated (from current standards) schizophrenic, possibly demented, individuals studied early in the last century are very different from the patients studied in this report, who are taking the latest antipsychotic medications, often can live independently, and are able to come to the lab by themselves either driving or taking public transportation. Thus both living environments as well as treatment medications of study populations differ substantially over time. Rosenzweig [19]was one of the first to demonstrate profound environmental effect on brain structure, and the remarkable plasticity of the brain in response to experience has been demonstrated on many levels [20].

The possible role of medication has been difficult to accurately determine. There appear to be differences in the influence of typical versus atypical antipsychotics in grey matter volume changes in schizophrenia [21], but reflections in total brain volume have not been reported. Older brain imaging studies tended to have less resolution because of slice thickness and related differences, although presumably such would only increase the variance in the data. Finally, might the "file drawer problem" [22] be playing a role, wherein only those studies reaching formal statistical significance get published, and the non-significant studies are relegated to the file drawer never to see the light of day? To the extent such a phenomena is present, the risk of meta-analyses being adversely impacted is also increased. Unfortunately there is yet no clear cut manner in which to examine the potential relevance of these issues.

With respect to VBR, we found SZ subjects had values significantly larger than controls, which is consonant with much of the published literature. One of the first observations in early imaging studies in SZ was evidence of larger ventricles, although again as with other variables the effect size seems to have diminished with time as Lewis has previously observed[4].

### Comparison of bipolar volumes

The brain volume of patients with bipolar disorder has been less intensively studied. In an earlier study Harvey, comparing brain volume of 26 subjects with bipolar disorder with 48 schizophrenics and 34 controls, found no difference between bipolars and controls, although the schizophrenic group had smaller volumes [23]. Friedman and colleagues studied cohorts of adolescents with schizophrenia and bipolar disorder compared to controls, and found evidence of decreases in brain volume when both patient groups were compared to controls, but the patient groups did not differ from each other [24]. Hoge et al reported a meta-analysis of 7 studies meeting criteria and examining cerebral volume in bipolar disorder, and concluded that there was no evidence supporting reduced brain volume in bipolar disorder[25]. A large meta-analysis published by McDonald et al 2004 systematically analyzed twenty six studies that investigated volumetric measurement on up to 404 bipolar patients. Their conclusions established that the volumes of most brain structures are preserved in bipolar disorder other than a noted association with right-sided ventricular enlargement [26]. Our bipolar findings are not at variance with the aforementioned studies.

### TBV/ICV ratio finding

**Figure 1:**
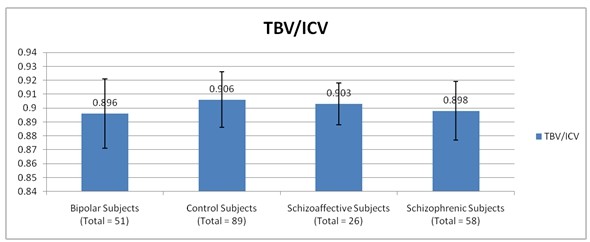
**BV/ICV ratio in the four subject groups**. Both bipolar and schizophrenic groups had a significantly lower ratio than control; schizoaffective subjects dif not differ from controls.

We found the TBV/ICV ratio to be decreased slightly but significantly in the SZ and BD (but not SAD) cohorts. These values are illustrated in Figure 1.

The finding is generally consistent with a small reduction in brain volume somewhere along the course of an illness, which, with preservation of the initial total ICV, leads to a decrease in the ratio of the two. The differences we found were in fact quite small - about 1% - or 12cc for a 1200 cc brain. Assuming the value is correct, its interpretation is uncertain, especially in light of little previous data supporting brain volume reductions in bipolar disorder, including this study.

### Comparison of schizoaffective volumes

The SAD group did not significantly differ from the normal control group on any variable. SAD is a diagnosis whose relationship to SZ or BD is not well understood. The proper categorization of SAD remains an enigma over seven decades after its initial description, and literature reviews to date have been able to contribute little clarity [27,28], and some investigators have questioned the existence of the syndrome [29]. Abrams and colleagues [30] provide an extensive recent review of the history, phenomenology, neuropsychological, physiological and genetic studies pertinent to SAD and conclude that the signs and symptoms of SAD cross conventional categorical boundaries between affective and other (schizophreniform) psychotic disorders, and that the study and treatment of SAD subjects would likely benefit from a dimensional rather than a categorical approach [30]. There is also an intrinsic confound in the diagnosis of SAD insofar as that a patients initially diagnosed as SZ may sometime later (perhaps years) develop and affective component (e.g. mania or psychotic depression) and thus the primary diagnosis may change to SAD. Once a SAD diagnosis is made however, it does not change to SZ.

Few published studies have examined SAD as an independent entity on the psychotic spectrum. Gruber and colleagues have suggested that relative preservation of articulatory rehearsal in verbal working memory in SAD as compared to SZ may constitute a neurocognitive endophenotype separating SAD from SZ [31]. Martin et al have suggested that there may be subdivisions within the SAD classification based upon variation in genetic and physiological measures relating to possible endophenotypes[32,33]. We have recently published MEG auditory evoked field based data supporting a biological difference between SAD and SZ possibly based upon relative preservation of neocortical inhibitory GABAergic interneuronal activity in SAD compared to SZ [34]. Such published findings along with our observations in this report would support further evaluations of SAD as a possible independent entity.

### Methodological issues

The volumes reported in this paper were collected over some period of time. To address the issue of reproducibility and possible drift over time, we randomly selected 17 of the brains extending over a time period of 10 years. The brain volume initially obtained by the rater (ER) was compared to the automated brain volumes computed by the Brain Extraction Tool (not available when the study started). An intraclass correlation coefficient (ICC) was computed between the two ratings on this series of 17 scans, and the ICC was .95, indicating both consistency among methods and lack of drift over time.

Finally, the specific methods utilized to estimate brain volume may well contribute significantly to overall volume estimates obtained from an experimental cohort and such methods have varied over time. For example earlier MRI studies frequently had relatively thick (e.g. 3-5 mm) sometimes non-contiguous slices which would contribute to variability of outcome measures. As computer power increased and image analysis software became more sophisticated, visually guided hand based cutting of structures, which is intrinsically very labor intensive, has been largely replaced by computerized image analysis with sophisticated algorithms based upon pixel intensity and rules of logic greatly facilitating automated analysis. Such methods lead to greater opportunity find specific brain regions associated with specific conditions at the expense of far greater statistical complexity as well as some uncertainty about accuracy of computer delineated structural volumes based primarily upon logic and pixel intensity. We believe this study may be the largest utilizing thin contiguous MRI slices and visually guided segmentation of the entire brain. Clearly such methodological differences may contribute to some of the variability of results reported in the literature, although precisely how much would be very difficult to estimate.

## Conclusions

In conclusion this study, although including a sizeable number of subjects, failed to demonstrate statistically significant differences in TBV between the three major groups of severe mental illness studied, although two groups (SZ and BD) demonstrated increased VBR, and the same two group demonstrated slight increases in TBV/ICV ratios. Although absolute raw data indicated brains in male SZ subjects were about 3% smaller than control brains, this failed to reach formal statistical significance. No findings in SAD subjects differed significantly from NC subjects, which along with other data discussed suggest further studies of SAD as a separate entity on the psychotic spectrum might be warranted. These findings should not, or course, be interpreted as supporting no difference in intrinsic brain structure in the psychotic disorders, as more refined neurohistological and computer derived neuroanatomical parcellation have suggested that such differences both exist and may be replicable, especially in SZ [35]. It may be some time however until such findings are useful in the definition of the single subject's pathology, treatment planning, and prognosis.

## Competing interests

None of the authors have interests that compete with the data presented and discussed in his manuscript.

## Authors' contributions

MR was Principal Investigator on the NIH grants that funded this research, and was responsible for the overall design and interpretation of the findings. ER personally segmented all MRI structures over the course of the study, and contributed to the literature review and discussion of how these findings relate to previously published findings by other laboratories. DC initiated and performed those computer analytic techniques used to clarify and improve resolution of several of the brain volume variable, and maintained the final data base contributing to the manuscript. PT supervised overall accuracy and comparability of imaging across the several MRI laboratories, and contributed to the final data analysis and manuscript preparation. DR was responsible for supervision of experimental design and final data analysis. ES was responsible for training in neuroanatomy and monitoring accuracy of image outlines. All authors have read and approved the final manuscript.

## Pre-publication history

The pre-publication history for this paper can be accessed here:

http://www.biomedcentral.com/1471-244X/10/79/prepub

## Supplementary Material

Additional file 1 **Addendum to methods**. Brief description of how dura was determined at the base of the brain in those posterior brain regions close foramen magnum. Click here for file

Additional file 2 **Total brain volume vs. age**. Scatter plot of total brain volume (ml) vs. age (years) Click here for file

Additional file 3 **VentricleBrainRatio vs. Age**. Scatter plot of ventricle brain ratio vs. age (years). Click here for file

Additional file 4 **Ventricular CSF volume vs. age**. Scatter plot of ventricular CSF volume (ml) vs. age (years) Click here for file

Additional file 5 **Brain ICV Ratio vs Age**. Scatter plot of Brain/ICV ratio vs. age (years). Click here for file
