# Supplementary material for: Brain size and brain/intracranial volume ratio in major mental illness
Source: BMC Psychiatry. 2010 Oct 11;10:79. doi: 10.1186/1471-244X-10-79 (PMC2958994; doi:10.1186/1471-244X-10-79)
Supplement: Additional file 1 — Addendum to methods. Brief description of how dura was determined at the base of the brain in those posterior brain regions close foramen magnum. [file 1471-244X-10-79-S1.DOC]

Addendum to Methods:

With respect to the boundaries of the ICV, the 3D acquisition as utilized well differentiates brain parenchyma from CSF based upon differences in pixel intensities in signal as derived from T1 weighted sequences employed. The extra axial boundaries are derived from analysis of the signal of the dura and diploic space permitting identification of the subdural contents. At the base of the brain efforts were made to carefully follow the dural signal, and if on certain slices there were interruptions in this signal and the dura could not be clearly identified for short distance, the rater used his knowledge of local neuroanatomy to estimate that line best connecting the dural signal between where it was lost and where it again resumed.
