# Supplementary figures and images for: Brain size and brain/intracranial volume ratio in major mental illness
Source: BMC Psychiatry. 2010 Oct 11;10:79. doi: 10.1186/1471-244X-10-79 (PMC2958994; doi:10.1186/1471-244X-10-79)

Total Brain Volume vs Age

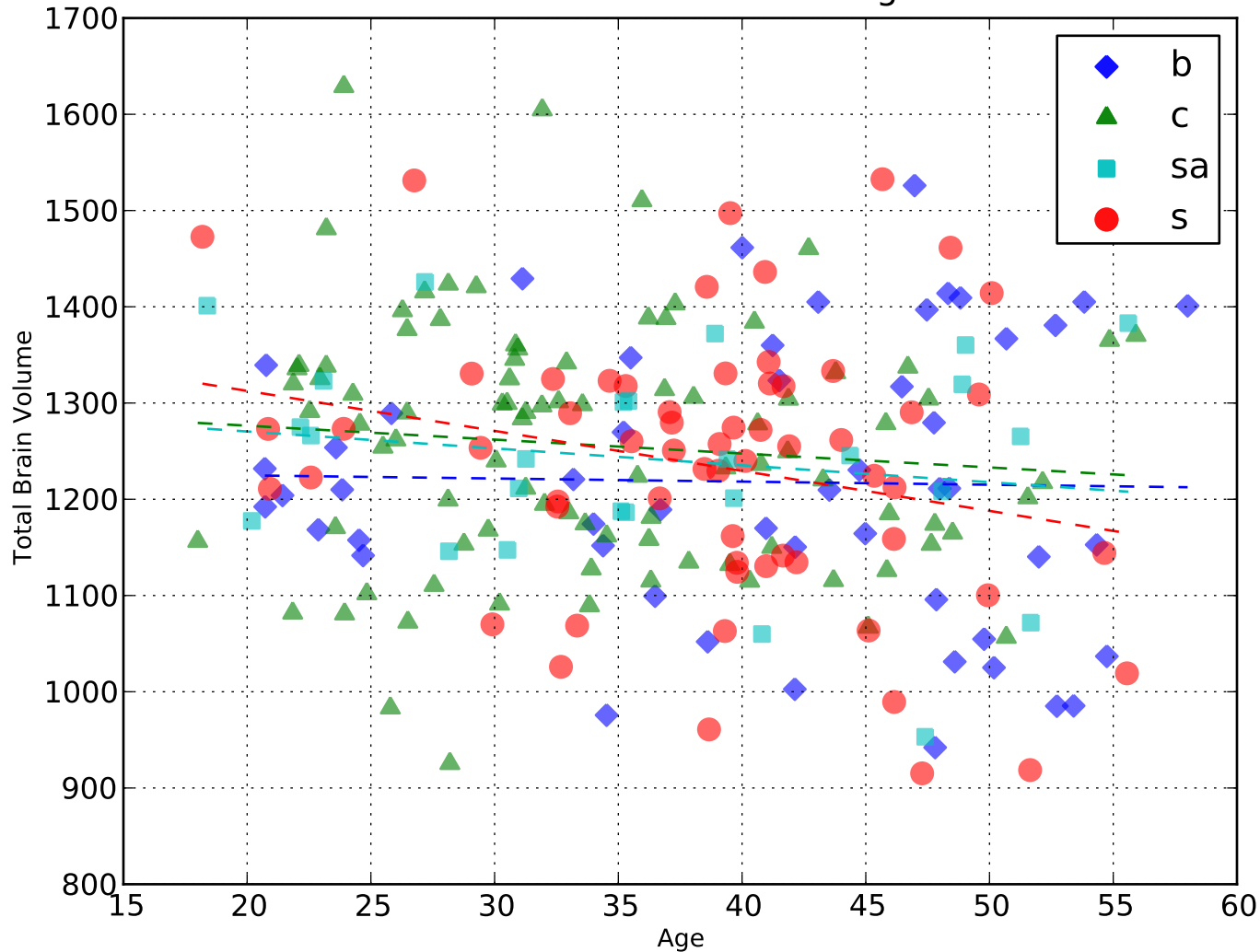

Supplement: Additional file 2 — Total brain volume vs. age. Scatter plot of total brain volume (ml) vs. age (years) [file 1471-244X-10-79-S2.PDF]

Ventricle Brain Ratio vs Age

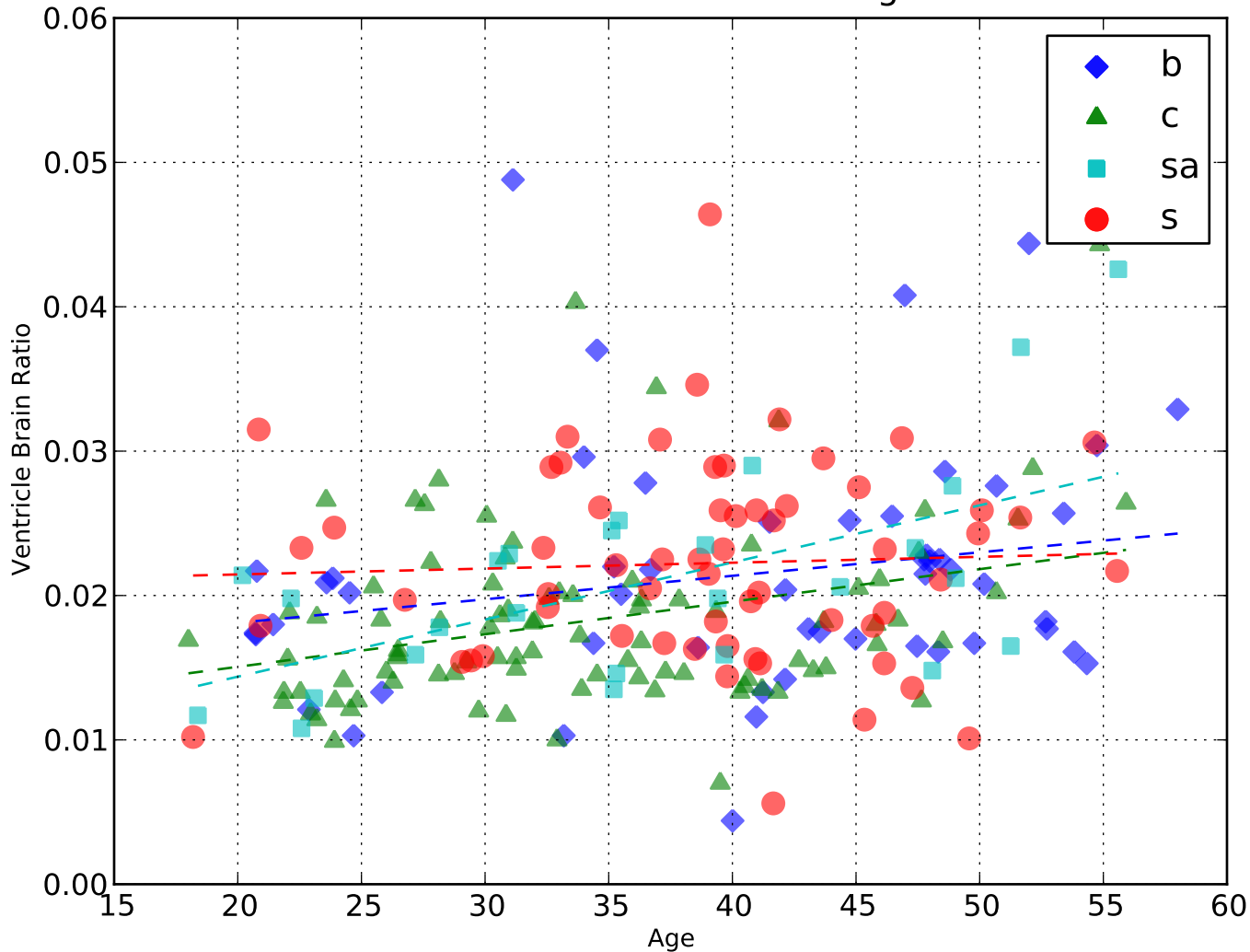

Supplement: Additional file 3 — VentricleBrainRatio vs. Age. Scatter plot of ventricle brain ratio vs. age (years). [file 1471-244X-10-79-S3.PDF]

Ventricular CSF vs Age

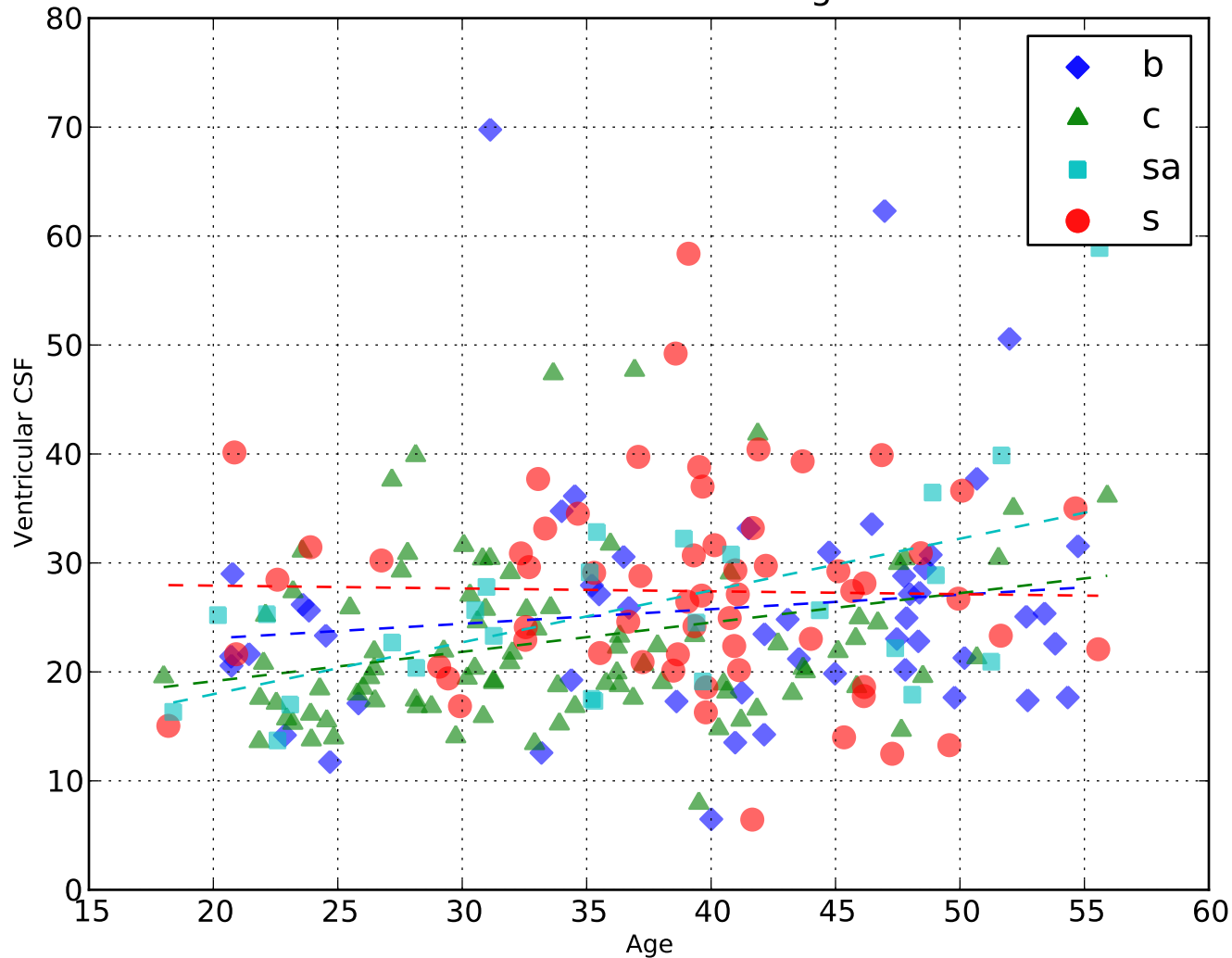

Supplement: Additional file 4 — Ventricular CSF volume vs. age. Scatter plot of ventricular CSF volume (ml) vs. age (years) [file 1471-244X-10-79-S4.PDF]

Brain ICV Ratio vs Age

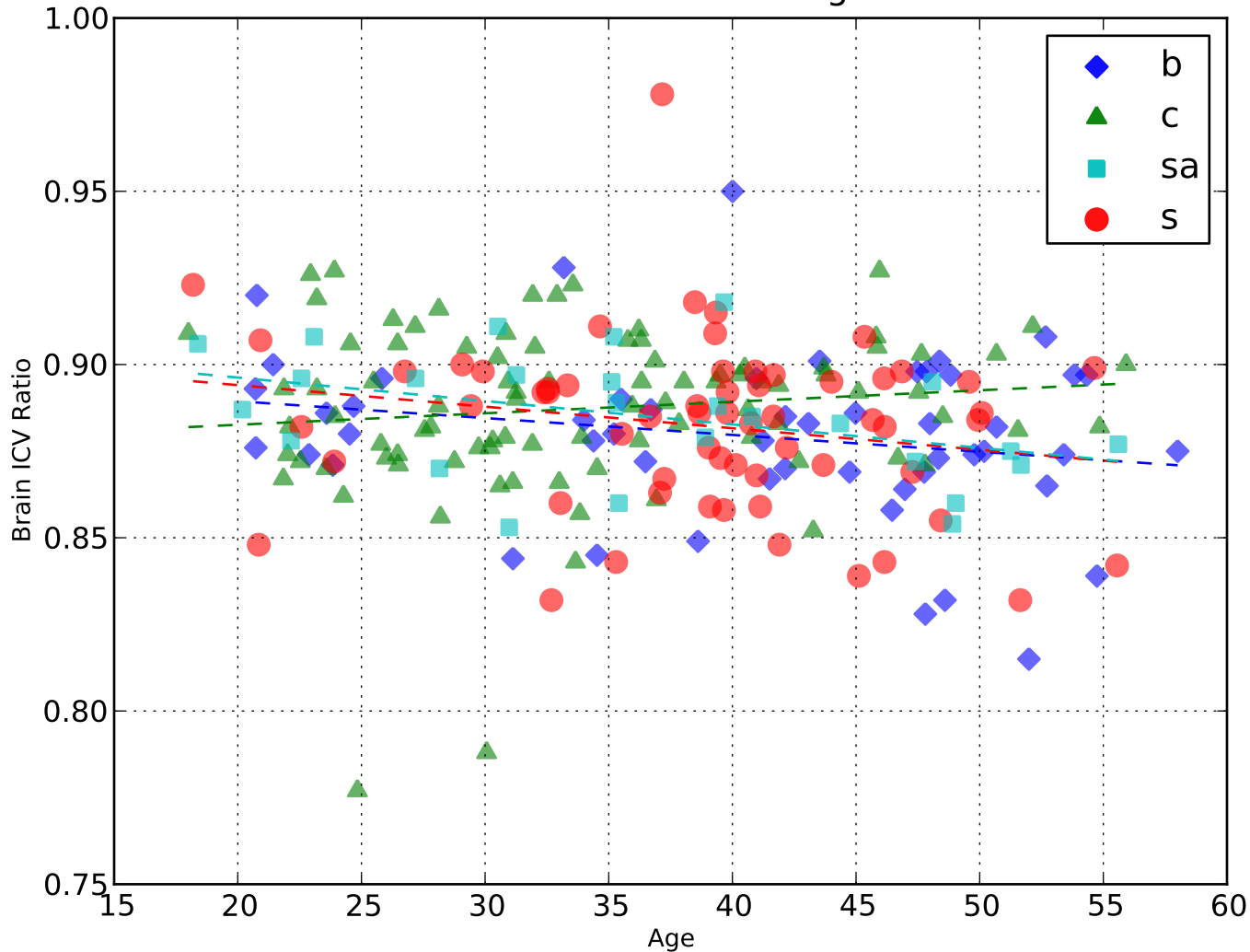

Supplement: Additional file 5 — Brain ICV Ratio vs Age. Scatter plot of Brain/ICV ratio vs. age (years). [file 1471-244X-10-79-S5.PDF]
